# Preoperative Variables Associated with Surgical Outcome for the Correction of Exodeviation

**DOI:** 10.3390/vision5020019

**Published:** 2021-04-23

**Authors:** Dominique Salh, Leah Walsh, Erik Hahn, Robert La Roche

**Affiliations:** 1 Eye Clinic, IWK Health Centre Children’s Site, 5980 University Avenue, Halifax, NS B3K 6R8, Canada; leah.walsh@iwk.nshealth.ca (L.W.); ehahn@dal.ca (E.H.); robert.laroche@dal.ca (R.L.R.); 2 Department of Ophthalmology & Visual Sciences, Faculty of Medicine, Dalhousie University, Halifax, NS B3H 4R2, Canada

**Keywords:** ophthalmology, strabismus, exotropia

## Abstract

The success rate of exodeviation surgery in existing literature has been shown to be variable. This study sought to determine the success rate of surgery for exodeviation in Atlantic Canada and determine variables associated with surgical outcome. A retrospective chart review was performed, considering patients who had been assessed and surgically treated for exodeviation at the IWK Health Centre between 2011–2018. This study included 176 subjects, aged 1–75 years. Preoperative variables were compared between subjects with successful versus unsuccessful surgical outcomes, using the chi square, Fischer’s exact test and binary logistic regression. A success rate of 43% was determined. Smaller preoperative deviation size at near and distance fixation, as well as the basic type classification were associated with successful operative outcome. Left eye acuity showed a statistically significant association with surgical success outcome. In conclusion, these findings compliment those of previous groups, suggesting exodeviation surgery outcome is variable. Our results add to a growing list of variables implicated in outcomes for these subjects. A smaller deviation preoperatively was associated with success in existing data and in this study, and these findings may suggest a potential role for basic subtype into future exodeviation literature.

## 1. Introduction

Exodeviations are outward eye turns that can present at any age, and affect about 1% of children under the age of 11 in western populations [1,2]. Intermittent exotropia is the most common type of outward eye turn [3,4]. Findings include one or either eye deviating outward some of the time, diplopia, headaches, photophobia/photalgia, closing or winking of one or either eye, or reduced binocular visual acuity [5,6,7]. Clinical assessment for this condition includes visual acuity, binocular functions, prism diopter (pd) measurements, assessments of control, classification of exodeviation and the presence of pattern deviations [1,8,9,10,11,12,13,14,15]. Both symptoms and assessment outcomes may be used to determine the need for surgery in this population and are important considerations prior to intervention [1,5,6,7,8,9,10,11,12,13,14,15]. Despite exodeviations being a commonly diagnosed type of strabismus, the management of exodeviation remains inconsistent [8,9,10,11].

Many patients with exodeviation are symptomatic and require surgical intervention [8,12,13,14,15]. However, the success of surgery for exodeviation has proven to be inconsistent in previous studies, where surgical success rates between 35.6–80.5% have been reported [16,17,18,19,20,21]. Clinical findings such as preoperative angle, stereoacuity, myopic refractive error, and the presence of a postoperative esodeviation have inconsistently been associated with successful surgical outcome [17,18,19,20,21]. The purpose of this investigation was to identify the success rate for exodeviation in a single population and determine the existence of preoperative variables associated with surgical outcome.

## 2. Materials and Method

### 2.1. Literature Search

MEDLINE, Google Scholar, and Science Direct were searched on 9 July 2018, using the search function with the following medical subject headings: exodeviation, intermittent exotropia, exotropia, exophoria, consecutive esotropia, surgery for exotropia, surgical outcomes of exotropia. No restrictions of language or date were applied. Electronic translation was used where literature was published in a foreign language. All valid studies and their references were considered in order to perform a thorough literature review.

### 2.2. Data Collection

This retrospective cohort study was performed between July 2018 to January 2019 at the IWK Health Centre in Halifax, Nova Scotia. This tertiary care center serves all of Atlanic Canada, where the population totals 2.3 million. Ethical approval was obtained from the IWK Research Ethics Board for the use of ophthalmology and orthoptic reports from preoperative assessments and follow up assessments following strabismus surgery. Patients unwilling to consent their health information to research were given the option to opt out at the time of their first clinic visit. Adult and child patients in this study were operated on by any one of five pediatric ophthalmologists, at a single site, for the surgical correction of an exodeviation between October 2011 and August 2018. Presurgical prophylaxis in the form of Betadine 5% was performed on all patients [22,23,24]. The amount of surgery performed depended on the angle of strabismus at 6m in primary gaze. The quantity of strabismus surgery was determined using established dosing tables (Table 1 and Table 2). The charts of 2014 patients were reviewed to determine surgical outcome rates. The null hypothesis predicted there would be no associations between success outcome and preoperative clinical characteristics.

### 2.3. Subject Selection

Patient charts were screened using a diagnostic code search for exodeviation and esodeviation, with an interest in selecting subjects with primary exodeviation before and after surgery, or consecutive esotropia after surgery. This revealed a total of 1920 charts for consideration of eligibility for inclusion. Manifest, intermittent, and exophoric deviations were all considered for inclusion. Subjects in this study underwent 1 of 5 different surgical procedures for the correction of the horizontal deviation: bilateral lateral rectus recessions, unilateral lateral rectus recession and medial rectus resection, unilateral lateral rectus recession and medial rectus tuck, unilateral lateral rectus recession only, or bilateral medial rectus resections.

To be included in this study, juvenile subjects (1–16 years) were required to have undergone cycloplegic refraction using 1% cyclopentolate, within 1 year prior to the operation date. Subjects requiring refractive correction for refractive error, including anisometropia, were required to have worn their prescription for at least three months prior to pre-operative orthoptic examination. Visually immature subjects with amblyopia underwent treatment prior to surgical consideration, until acuity equalized or until the amblyopic eye did not improve for three consecutive cycles of follow up appointments. All included subjects had normal fundus exams, as per examination by a fellowship trained pediatric ophthalmologist. Although this investigation involved subjects of all ages, subjects had to have reliable, monocular LogMAR visual acuity recorded prior to their operation to meet the inclusion criteria. Subjects with a history of any previous orbital or ocular surgery, the presence of dissociated vertical deviation, manifest or latent nystagmus, or the presence of any neurological or mechanical abnormalities that are consistent with congenital exotropia or other abnormalities of ocular alignment were excluded.

For the determination of recurrence rate, 176 subjects met the inclusion criteria, thus their postoperative status was considered. For the statistical comparison of successful and unsuccessful groups, we required a random sample of only 130 subjects across both groups to achieve adequate power, β = 0.80 and *p* = 0.05. An analysis was performed using G* Power statistical calculation software [25]. Sixty-five subjects with both successful or unsuccessful surgical outcomes were randomly sampled, for a total of 130 subjects for statistical analysis. All 176 patients were not included in the final statistical analysis due to ethical considerations for patient data collection, where only 130 charts would be required to determine a significant effect according to the power analysis. Outcomes of clinical examinations from pre- and postoperative visits were recorded. Preoperative outcomes were measurements taken no more than one week before the surgical date. Postoperative outcomes were derived from assessments at least 3 months after the operation date, and again at the most recent follow up exam at the time of data collection.

### 2.4. Defining Success

To maintain consistency with the majority of pre-existing literature, alignment criteria was used to determine surgical success [16,17,19,21]. Any horizontal deviation that approached fusion range, not exceeding 10 pd in primary position measured at near and distance fixation, was classified as successful. If a postoperative deviation was greater than 10 pd at either near or distance fixation, the outcome would be categorized as unsuccessful. Measurements from the initial postoperative visit and the most recent follow up exam at the time of data collection were considered.

### 2.5. Variables of Interest

Given the inconsistent nature of variables associated with surgical outcomes from previous studies, this investigation aimed to capture any preoperative variables that could relate to clinical presentation or symptomatology. Some of these variables were recorded to allow for the control of confounding variables during statistical analyses. The main outcome variables for consideration in this study included measures of visual acuity, sensory fusion, ocular alignment, and details regarding alignment characteristics. A list of collected variables can be found in Table 3.

### 2.6. Data Collection

All subjects meeting the inclusion criteria formed a large sample to determine an overall percentage of surgical success in exodeviations at the IWK Health Centre. After determining success outcomes, 130 subjects from either the unsuccessful or successful groups were randomly sampled. Subjects with over-corrections greater than 10 pd of esodeviation were not included in the random sampling, because this population did not reach statistical power to enable inclusion into the analysis.

Clinical data was acquired during perioperative orthoptic and ophthalmologic examinations. Both the Early Treatment Diabetic Retinopathy Study (ETDRS Triple) and Lea Hyvarinen (LH Triple) Symbols (CSV-1000, Vector Vision, Dayton, OH, USA) charts were used for the measurement of visual acuity in this investigation. For the younger subjects, either verbal naming of indicated letter or matching shapes to a handheld card during acuity testing was acceptable. The Sloan Letter Near Card^®^ (Catalog number: 72500, Good-lite Co., Elgin, IL, USA, Precision Vision, Woodstock, IL, USA) or (LH) Symbols near card (Catalog number: 250800, Good-lite Co., Elgin, IL, USA; Precision Vision, Woodstock, IL, USA) were used for near visual acuity. Visual acuity was scored according to the logMAR score corresponding to the acuity of each eye.

Deviation size was acquired using the alternate prism cover test where possible, or using the Krimsky, or modified Krimsky, method where reliable prism cover test measures where not obtainable. Duane’s clinical classification was applied to cover test measurements using a maximum difference of 10 pd of near-distance disparity to assign classification. Pseudo-divergence excess subtype was established when the deviation at near increased to within 10 diopters of the distance deviation in primary position, after applying +3.00D lenses and an accommodative target. Accommodative convergence to accommodation ratio (AC/A) was calculated using the gradient method, and measurements were taken using an accommodative target at 1/3 m.

### 2.7. Statistical Analysis

Descriptive statistics characterized the demographic trends within the sample. A chi-squared analysis was used to evaluate potential relationships between postoperative outcome and categorical data for variables with 3 or more subcategories. If the assumptions of this analysis were violated per IBM SPSS statistics for Mac v. 25 (IBM Corp., Armonk, NY, USA), the Fischer’s exact test was used instead. To compare means of preoperative scale variables between subjects with successful and unsuccessful surgical outcomes, a binary logistic regression was performed. The regression was used in lieu of a biserial correlation, to determine an association between surgical outcome and continuous variables, where assumptions of other analyses had been violated [26]. This analysis was chosen due to the binary categorical nature of the outcome variable: success versus lack of success. Due to the various types of variables assessed in this study, for example continuous versus categorical, one type of statistical test could not be applied broadly throughout the analysis.

## 3. Results

One hundred seventy-six subjects were enrolled. Of these, 91 subjects were found to be unsuccessful and 76 subjects were successful, within 10 diopters of orthotropia. The remaining 9 subjects were overcorrected with esodeviations exceeding 10 pd at near or distance postoperatively. Some of these subjects required further surgery or alternative treatment such as bifocal lenses and, as mentioned previously, constituted too small of a sample for statistical analysis. Overall, this information suggests a success rate of 43%, a 52% recurrence rate, and a 5% overcorrection rate for the surgical correction of exodeviations. The overcorrected subjects were disregarded from further analysis for reasons explained previously.

Of the 130 randomly selected subjects, 21 subjects had manifest exotropia, 107 subjects had intermittent exotropia, and 2 subjects had symptomatic exophoria. Each group consisted of 34 females and 31 males, which unintentionally generated a sex-matched sample (Table 3). Preoperatively, 83 subjects had basic type deviations, 30 had pseudo divergence excess, 3 had true divergence excess, and 14 had convergence insufficiency subtype deviations. In consideration of surgical procedure, 56 underwent unilateral lateral rectus recession and medial rectus resections, 28 underwent bilateral lateral rectus recessions, 38 underwent unilateral lateral rectus recessions and medial rectus plication, 5 underwent unilateral lateral rectus recession, and 3 underwent bilateral medial rectus resections. This study included patients with manifest, intermittent, and phoric deviations, while excluding exodeviations of congenital origin. Both patients with phoric control had successful surgical outcomes, 8 out of 22 (36%) manifest deviations had successful outcome, and 55 of 106 (52%) patients with intermittent exotropia had successful outcomes.

A total of 6 preoperative variables were significantly associated with surgical outcome, suggesting that the null hypothesis could be rejected (Table 4). These variables include Duane’s clinical classification (basic type), near deviation, distance deviation, target angle of surgery, deviation at 1/3m with +3.00 D lenses, and left eye acuity.

## 4. Discussion

In this investigation, we addressed the recurrence rate of exodeviation surgeries in subjects aged greater than 1 year up to 75 years. In this study 82% of subjects were 16 years and younger; the inclusion of both pediatric and adult populations within a single analysis has been studied previously, and age was not significantly related to surgical outcome [27]. The present analysis revealed a success rate of 43%, and this number is in keeping with other studies that use a similar criteria for success [16,17,18,19,20,21]. Further, we assessed relationships between surgical outcome and preoperative clinical characteristics.

Our findings partially complimented those of previous researchers, where preoperative angle of deviation in the present analysis was significantly associated with surgical outcome [17,18]. In a study by Zou et al., preoperative deviation is predictive of a successful outcome through a predictive modelling analysis, by contrast [17]. Previous research also shows reduced visual acuity is correlated with poorer surgical outcome, which was also found in the present investigation [28]. Although, left eye and not right eye acuity were statistically associated with surgical outcome in this study, this may be of limited clinical importance. Mean LogMAR left eye acuity between successful and unsuccessful groups were 0.4 Log units different at approximately 6/7.5 vision. This is compared to right eye acuity, which reveals a 0.3 Log unit difference between successful and unsuccessful groups and is not statistically significant. Presently, we are unable to determine a significant association with refractive error or stereoacuity, as previous researchers have found [17,18].

Other findings of this study are not in keeping with the existing literature. Our results did not replicate the findings of Bae et al., who report greater postoperative success in patients with pseudo-divergence excess exotropia (70.2%), than for basic type (46.3%) or true divergence excess (28.6%) in a sample of 342 patients from Korea at 1, 3, 6, and 12 month follow up [15]. Their findings suggest that pseudo-divergence excess patients also show lower recurrence rates, based on the need for subsequent surgeries [15]. Aside from genetic differences between populations based in Asia versus Atlantic Canada, it is hard to identify a reason for this difference in findings between those of Bae at al., and those of the present study. Surgical approach between study groups for pseudo-divergence excess subjects, or the method for determining alignment postoperatively in these patients may be considered as contributing to this difference.

One additional finding of this study was the association between left eye acuity and surgical outcome. Unsuccessful subjects had on average, 0.5 LogMAR lines of acuity less than successful subjects. Although this variable was statistically significant, the clinical difference between 0.9 LogMAR (success group mean), and 0.14 LogMAR (unsuccessful group mean) may be negligible. Amblyopia was not significantly related to success in the present analysis as per Table 4.

Regarding associations between preoperative characteristics and surgical outcomes, it is difficult to draw comparisons between this investigation and previous ones. The present investigation differed from others in terms of statistical analyses performed, where the present study did not calculate statistical risk factors [19,21]. Due to the existence of missing data for variables of clinical interest such as AC/A ratio, preoperative deviation at 20 ft, or following a 45 min patch test, it is difficult to determine whether a lack of significance in this study is due to a nonexistent association, or lack of statistical power. Comparisons between the present investigation and others are also limited by the population of interest, with respect to age range and control of the exodeviation.

Design Limitations

This investigation was limited significantly by its retrospective design, including unstandardized testing protocols, and the inclusion of five surgeons for the treatment of all patients. Further, the prism cover test measurements and visual testing outcomes were collectively obtained by a team of 8 orthoptists. Surgical procedure was at the discretion of the operating surgeon, and perioperative management was also under physician discretion. There may be considerable similarities between the orthoptists and surgeons, respectively, who work in the same center, partially limiting inter-individual variability in the study. The limitations of this design should support the need for future prospective studies that attempt to control the previously mentioned confounding variables.

The consideration of follow up schedules when analyzing the outcomes and follow-up management for patients following surgery for exodeviation. The present study assessed outcome rates based on measurements taken during the 3-month postoperative visit. These findings compliment the findings described by Oh and Hwang, where early postoperative esodeviation is the only predictor of success [21]. Future directions may exclusively assess later postoperative clinical findings in a similar study, to be compared with most recent follow up clinical outcomes, which are not considered in this study due to scope of objectives.

## 5. Conclusions

A smaller preoperative deviation at either near or distance fixation was shown to be associated with successful surgical outcomes in this investigation, which included patients of all ages with manifest, intermittent, or phoric exodeviations [17,18]. The replication of these findings across studies may provide merit to this finding. The findings of this investigation also highlighted a potential role for Duane’s basic type clinical classification into the existing body of literature as potentially being both associated with successful outcome. This encourages the possibility of future, prospective studies considering this relationship.

## Figures and Tables

**Table 1:** Surgical table for asymmetrical two muscle surgery.

Exodeviation Size (Prism Diopters)	LR Recession AND	MR Resection/Plication
15 diopters	4	3
20 diopters	4	4
25 diopters	6	4.5
30 diopters	6.5	5
35 diopters	7.5	5.5
40 diopters	8	6
50 diopters	9	6
60 diopters	10	6
70 diopters	10	7
80 diopters	12	9

**Table 2:** Surgical table for symmetrical two muscle surgery.

Exodeviation Size (Prism Diopters)	LR Recess OU OR	MR Resect OU
15 diopters	4.5	3
20 diopters	5.5	4
25 diopters	6	4.5
30 diopters	7	5
35 diopters	8	5.5
40 diopters	9	6
50 diopters	10	-

LR = Lateral Rectus; MR = Medial Rectus; OU = Both eyes.

**Table 3:** Mean values describing patient demographics and clinical presentation from successful and unsuccessful groups. (pd= prism diopters).

Variable	Successful Group (*n* = 65)	Unsuccessful Group (*n* = 65)
Sex (F:M)	34/31	34/31
Age at Surgery	14.98 Range 1–67	11.59 Range 1–75
Preop angle near (pd)	21.31 Range 6–63	31.92 Range 4–183
Preop angle distance (pd)	24.46 Range 10–58	32.25 (10–141)
Stereoacuity	146	215.2
Visual Acuity LOGMAR (right)	0.12	0.13
Visual Acuity LOGMAR (left)	0.9	0.14
Duane’s Classification	Basic (*n* = 49) Pseudo-divergence excess (*n*= 13) Divergence excess (*n* = 1) Convergence insufficiency (*n* = 2)	Basic (*n* = 34) Pseudo-divergence excess (*n* = 17) Divergence excess (*n* = 2) Convergence insufficiency (*n* = 12)
Follow up (weeks)	84.15 (8–339)	111.35 (8–344)

**Table 4:** The association between surgical outcome and preoperative characteristics.

Variable	Association Statistic	Significance Value (*p*)	*n* Value
Duane’s Pre Op Cramer’s V	10.936 0.287	0.007 * σ 0.010 *	130
Near PreOp Angle	7.751	0.005 * ψ	130
Distance PreOp Angle	9.447	0.002 * ψ	130
Target Angle in Surgery	7.107	0.008 * ψ	110
+3.00D Deviation Pre Op	7.383	0.007 * ψ	119
AC/A Ratio (using +3.00D measurement)	0.394	0.530 ψ	119
Age Surgery	0.974	0.324 ψ	130
Stereopsis Near	0.548	0.459 ψ	129
Stereopsis Distance	1.017	0.301 ψ	121
Control Subtype Pre Op	2.072	0.391 ω	130
Dis VA RE	0.073	0.787 ψ	130
Dis VA LE	3.946	0.047 * ψ	130
Interocular Difference VA	1.023	0.312 ψ	130

σ = Chi Squared Test, ω = Fischer’s Exact Test, ψ = Binary Logistic Regression, * = Statistical significance.

## Data Availability

Not applicable.
